# Social support, perceived risk and the likelihood of COVID-19 testing and vaccination: cross-sectional data from the United Kingdom

**DOI:** 10.1007/s12144-021-01681-z

**Published:** 2021-04-08

**Authors:** Rusi Jaspal, Glynis M. Breakwell

**Affiliations:** 1 Department of Psychology, Nottingham Trent University, Nottingham, NG1-4FQ UK; 2 Department of Psychology, University of Bath, Bath, UK; 3 Institute of Global Health Innovation, Imperial College, London, UK

**Keywords:** COVID-19, Testing, Vaccination, Social support, Self-efficacy, Perceived risk

## Abstract

Two samples of 227 and 214 adults completed surveys of social support, perceived risk of COVID-19 and COVID-19 preventive activity – in Study 1 likelihood of testing was examined and in Study 2 likelihood of both testing and vaccination were examined during the COVID-19 pandemic in the United Kingdom. Path analysis showed, in Study 1, that access to help (as an indicator of social support) had a direct effect on likelihood of testing and indirect effects through self-efficacy, perceived risk and preventive behavior; and, in Study 2, that neighborhood identification (as an indicator of social support) had a direct effect on likelihood of testing and indirect effects on likelihood of both testing and vaccination through the mediators of strength of social network, loneliness, perceived risk of COVID-19, and preventive activity. Both studies suggest that level of social support (conceptualized in different ways) is an important determinant of COVID-19 testing and Study 2 shows it is also a determinant of likelihood of vaccination. As resurgences of COVID-19 occur, it will be necessary to monitor the likelihood of COVID-19 testing and vaccination behaviors and, especially, to promote confidence in the latter in individuals with decreased access to social support.

## Introduction

Since its initial clinical observations in Wuhan, China in December 2019, COVID-19 has been designated a pandemic and most nations in the world have struggled to contain the virus. In the United Kingdom (UK), there have already been significant resurgences of the disease after the initial outbreak and lockdown in March 2020. In addition to activity to prevent one’s own risk of infection, such as adherence to social distancing and the wearing of a face covering in public, two behaviors will be key to curbing the incidence of COVID-19 in the long term – testing for and vaccination against the disease. In this article, a distinction is specifically made between general preventive behavior and the likelihood of testing and vaccination. There is now considerable research into public acceptability of testing and vaccination in relation to both COVID-19 and other diseases, which suggests that these are complex social psychological issues (e.g., Bertin et al., 2020; Vandrevala et al., 2020). Perceived risk of infection and willingness to engage in other preventive activity are generally associated with increased acceptability of testing and vaccination (Blanchard-Rohner et al., 2020). There is emerging evidence that social support is an important determinant of preventive activity in relation to COVID-19 (Mols, 2020). However, studies have not yet focused on the impact of social support, over and above perceived risk of infection, on testing and vaccination likelihood. This article presents data from two cross-sectional studies modelling the impact of social support (conceptualized here in Study 1 as having help at hand during times of need, and in Study 2, in terms of neighborhood identification) on the likelihood of COVID-19 testing and vaccination in the UK.

### COVID-19 Testing and Vaccination

In order to control COVID-19, it is essential to understand what influences public acceptance of testing and vaccination. In addition to considerable research on testing in other disease contexts, such as that of HIV (Deblonde et al., 2010), there is now emerging work on public acceptance of COVID-19 testing. Hall et al. (2020) found that 69% of their participant sample would be more likely to test for COVID-19 if the test were performed at home compared to in a drive-through setting. Public acceptance of COVID-19 testing in clinical settings is considerably lower – 60% (Siegler et al., 2020). In their survey in the UK, Vandrevala et al. (2020) found that concerns about the impact of COVID-19 on self and family were positively associated with willingness to test.

There are many competing social representations of vaccination, which in part determine its acceptability in the general population (Bish et al., 2011; Larson et al., 2014). Feleszko et al. (2020) found that 28% of their Polish sample would refuse to get vaccinated against COVID-19 if a vaccine were available. In their survey study of 991 US adults, Fisher et al. (2020) found that 31.6% were unsure about being vaccinated and that 10.8% did not intend to do so. Neumann-Böhme et al. (2020) identified several socio-demographic groups at especially high risk of COVID-19 vaccine hesitancy in various European countries; they also examined the specific reasons provided by respondents for their stance on vaccination. Many related to beliefs about the efficacy and effects of the vaccine. Similarly, the endorsement of both COVID-19 conspiracy beliefs and general conspiracy beliefs was inversely associated with vaccine acceptability in two French samples (Bertin et al., 2020). The role of social support in relation to likelihood of testing and vaccination has not yet been studied.

### Social Support and COVID-19 Prevention

According to the social cure perspective (Jetten et al., 2012), identification with meaningful social networks, such as one’s neighborhood, is psychologically beneficial largely due to its ability to provide social support. During the era of social distancing, social support has come to be derived in many different, ‘COVID-safe’ ways (Nerlich & Jaspal, 2021). In the present studies, four aspects of social support, namely access to social support in times of difficulty, strength of neighborhood identification, strength of social network, and extent of loneliness, are examined. The purpose of using these distinct aspects was to generate evidence of the relevance of social support, as a multifaceted construct, in relation to likelihood of testing and vaccination. Study 1 focuses on the effects of access to social support. Study 2 explores the effects of neighborhood identification. Since previous research has shown that neighborhood identification is positively associated with strength of social network (Moyano-Díaz & Mendoza-Llanos, 2021), which, in turn, is negatively associated with loneliness (Rolandi et al., 2020), these two aspects of social support are included in Study 2.

It has been found that social support (when derived from a meaningful social group membership) may promote feelings of self-efficacy in relation to health behaviors advocated by that social group (Guan & So, 2016). This may be especially relevant in collectivist societies in which the family is central to identity (Wang et al., 2021). Self-efficacy has been shown to predict preventive activity but it is possible that perceived risk mediates this relationship (Imai et al., 2020). There is also emerging evidence that social support is an important precursor of engagement in preventive activity during the pandemic (Mols, 2020). In fact, Stickley et al. (2020) found that loneliness (an indicator of decreased social support) was inversely associated with the likelihood of hand washing, wearing a face covering and social distancing when in public.

A strong connection with relevant social networks, such as one’s neighborhood, can provide access to risk and prevention information (Finnegan et al., 1993), enabling people to appraise their risk effectively but also to take preventive action (Jaspal & Lopes, 2020). However, loneliness (as a particular state reflecting decreased social support) has been shown to be associated with an over-estimation of one’s own risk (Okruszek et al., 2020). Overall, having a strong social network – particularly at a time of enforced self-isolation due to COVID-19 – appears to be psychologically beneficial and conducive to better behavioral outcomes, including engagement in preventive activity. Conversely, decreased access to a social network can result in a psychological state of loneliness, which itself may be disempowering in terms of information acquisition, self-efficacy and action (Cacioppo & Hawkley, 2009; Stickley et al., 2020).

As an indicator of social support (Mair et al., 2010), neighborhood identification (Fong et al., 2019a, 2019b) refers to the extent to which individuals perceive their neighborhood as an element of their identity – it can be assessed in terms of the level of importance one appends to one’s neighborhood, how happy one feels about being a resident of it, how fulfilled one feels by it, and the extent to which one’s neighborhood affiliation guides one’s behavior and self-presentation. Neighborhood identification may facilitate access to a meaningful social network, mitigate loneliness and thus empower individuals to acknowledge risk and to engage in potentially challenging preventive activity. It may constitute a source of social norms that prompt individuals to engage in certain behaviors (in this case, preventive activity) through social influence and stigmatization of behavioral non-compliance (Guan & So, 2016). Yet, neighborhood identification must be viewed as part of a system of social psychological factors, including perceived risk of infection and existing engagement in preventive activity, which potentially determine one’s likelihood of testing and vaccination. Thus, it was hypothesized that, as a form of social support, neighborhood identification will be associated with strength of social network, decreased loneliness and increased likelihood of testing for and vaccination against COVID-19.

Given the multi-faceted nature of the social support construct, two studies were conducted - one in which social support was indexed in terms of personal access to help and another in which it was indexed in terms of neighborhood identification. Loneliness was treated as a psychological state and measured as a separate variable to assess its relationship to neighborhood identification and strength of social network.

### Perceived Risk of COVID-19 and Preventive Activity

Perceived risk has been shown to influence behavior, especially in relation to hazards, such as disease (Clifton et al., 2016; Kahle et al., 2018). Research also shows that perceived risk of COVID-19 is positively associated with both willingness to be tested for and vaccinated against the disease (Blanchard-Rohner et al., 2020; Reiter et al., 2020).

Yıldırım et al. (2020) found that perceived risk of COVID-19 was a significant predictor of engagement in preventive behaviors. It is also likely that engaging in COVID-19 preventive activity (such as wearing a face mask and adherence to social distancing), as a self-care behavior, will also be associated with increased likelihood of testing and vaccination. However, it must also be noted that social representations of preventive behaviors vary by cultural context – in Eastern Asian countries, for instance, wearing a face covering was a social norm even before the pandemic (Jaspal & Nerlich, 2020; Wang et al., 2020). Furthermore, risk appraisal is based on many distinct factors, including social representations and social norms, which in turn will shape willingness to adopt preventive behaviors (Breakwell, 2014).

In the context of HIV, it has been found that people who engage with HIV testing are also more likely to endorse other novel approaches to preventing infection, such as pre-exposure prophylaxis (Jaspal et al., 2019). This could be attributed to an overall ‘prevention norm’, which motivates members of a collective to endorse and comply with particular preventive behaviors that are socially represented as being desirable or even necessary in a risk context. Accordingly, it was hypothesized that people who already engage in COVID-19 preventive activity will be primed to undertake additional activity, such as testing and vaccination, to reduce their own risk of infection.

### Objectives

Two cross-sectional studies were designed to examine the associations between social support (defined in Study 1 as personally having help at hand and in Study 2 as feeling identified with one’s neighborhood), perceived risk, preventive behavior and likelihood of testing and vaccination. In Study 2, the effects of social network and loneliness were also tested. It is noteworthy that testing and vaccination were differentiated from other forms of COVID-19 preventive behaviors.

## Study 1

The aim of Study 1 was to examine associations between social support (conceptualized as having help at hand in times of need), self-efficacy, perceived risk of COVID-19, preventive behavior (e.g., wearing a face mask, keeping a physical distance of at least two meters from others and so on) and the likelihood of testing for COVID-19. The following hypotheses were tested:

1. Social support will be positively associated with likelihood of testing after the effects of perceived risk and level of other preventive activity are taken into account.
2. Social support will be positively associated with self-efficacy.
3. Self-efficacy will be positively related to perceived risk of COVID-19.
4. Perceived risk of COVID-19 will be positively associated with both preventive activity and likelihood of testing for COVID-19.
5. Preventive activity will be positively associated with likelihood of testing for COVID-19.

### Method

#### Ethics

This project received ethics approval from Nottingham Trent University’s College of Business, Law and Social Sciences Ethics Committee. Participants provided electronic consent to participate, were debriefed and thanked for their time.

#### Participants

Two-hundred and twenty-seven individuals were recruited on Prolific, an online participant recruitment platform, to complete an online survey on COVID-19 preventive activity and testing during the COVID-19 pandemic in the UK. Participants were recruited on 14 August 2020 when testing was widespread and available. There were two sole eligibility criteria: (1) being aged 18 or over, and (2) being a resident in the UK. See Table 1 for a full summary of the socio-demographic characteristics of the participant sample in Study 1.

**Table 1:** Socio-demographic characteristics of the sample in Study 1

**Gender**	**Male**	**Female**					
	*N* = 72 (31.6%)	*N* = 154 (67.5%)					
**Ethnicity**	**White British**	**Indian**	**Pakistani**	**Bangladeshi**	**African**	**Caribbean**	**Mixed**
	*N* = 116 (50.9%)	*N* = 42 (18.4%)	*N* = 20 (8.8%)	*N* = 12 (5.3%)	*N* = 19 (8.3%)	*N* = 11 (4.8%)	*N* = 8 (3.5%)
**Income**	**<£10,000**	**£10,000 to £19,999**	**£20,000-29,999**	**£30,000-39,999**	**£40,000-49,999**	**£50,000-59,999**	**£60,000>**
	*N* = 61 (26.8%)	*N* = 49 (21.5%)	*N* = 51 (22.4%)	*N* = 39 (17.1%)	*N* = 14 (6.1%)	*N* = 3 (1.3%)	*N* = 10 (4.4%)
**Employment**	**Employed**	**Self-employed**	**Furloughed**	**Student**	**Retired**	**Unemployed**	
	*N* = 118 (51.8%)	*N* = 17 (7.5%)	*N* = 10 (4.4%)	*N* = 59 (25.9%)	*N* = 5 (2.2%)	*N* = 16 (7%)	
**Education**	**GCSE/O-Levels**	**A−/ AS-Levels**	**Undergraduate**	**Postgraduate**	**Apprenticeship**	**Other**	
	*N* = 21 (9.2%)	*N* = 65 (28.5%)	*N* = 99 (43.4%)	*N* = 38 (16.7%)	*N* = 1 (.4%)	*N* = 3 (1.3%)	
**Previous COVID-19 test**	**Yes** *N* = 26 (88.2%)	**No** *N* = 201 (88.2%)					

#### Measures

Participants were asked to indicate their age, gender, citizenship, ethnicity, level of education, employment status, income and whether or not they had tested for COVID-19 previously.

##### Social Support: Access to Help

An adapted version of the Interpersonal Support Evaluation List (Cohen et al., 1985) was used to measure access to social support in terms of having help at hand during times of difficulty. The adapted scale consists of 4 items (e.g., ‘When I need suggestions on how to deal with a personal problem, I know someone I can turn to.’) Items were measured on a 4-point scale (1 = definitely false to 4 = definitely true). The scale manifested excellent reliability (α = .82).

##### Self-Efficacy

The Generalized Self-Efficacy Scale (Schwarzer & Jerusalem, 1995) was used to measure self-efficacy. The scale consists of 10 items (e.g., ‘I am confident that I could deal efficiently with unexpected events.’) Items were measured on a 5-point scale (1 = strongly disagree to 5 = strongly agree). The scale manifested excellent reliability (α = .89).

##### Perceived Risk of COVID-19

The COVID-19 Own Risk Appraisal Scale (CORAS) (Jaspal et al., 2020) was used to measure perceived risk of COVID-19. The scale consists of 6 items (e.g., ‘I am sure I will NOT get infected with COVID-19.’) Items were measured a 5-point scale (1 = strongly disagree to 5 = strongly agree). The scale manifested excellent reliability (α = .86).

##### COVID-19 Preventive Activity

The COVID-19 Preventive Behavior Index (CPBI) (Breakwell et al., 2021) was used to measure likelihood of engaging in COVID-19 preventive activity. The scale consists of 10 items (e.g., ‘How likely is that, during the COVID-19 outbreak, that you will use a facemask when you leave your home?’) Items were measured on a 5-point scale (1 = extremely unlikely to 5 = extremely likely). The scale manifested acceptable reliability (α = .75).

##### Likelihood of Testing for COVID-19

The item ‘How likely is that, during the COVID-19 outbreak, that you will seek to get tested for the virus?’ was measured on a 5-point scale (1 = extremely unlikely, 5 = extremely likely).

### Results

First, independent samples *t*-tests were performed to examine differences between participants who had never been tested (*N* = 201) vs. those who had (*N* = 26) in social support, perceived risk of COVID-19, COVID-19 preventive activity, and likelihood of COVID-19 testing in the future. There were no significant differences (*p* > 0.05) and, thus, all subsequent analyses were carried out on the entire sample (*N* = 227).

#### Descriptive Statistics

Table 2 provides an overview of the descriptive statistics for Study 1.

**Table 2:** Descriptive statistics for the key variables in Study 1

Continuous variables	Mean	SD	Minimum	Maximum
Age	31.18	10.85	18	65
Social support: access to help	12.37	2.96	4	16
Self-efficacy	36.26	5.87	15	50
Perceived risk of COVID-19	18.09	4.66	7	28
COVID-19 preventive activity	40.38	5.87	23	50
Likelihood of testing for COVID-19	3.67	1.16	1	5

#### Correlations

Table 3 provides a summary of the correlations between the main variables in Study 1. Social support was positively associated with both self-efficacy and likelihood of testing for COVID-19. Self-efficacy was negatively associated with perceived risk of COVID-19. Perceived risk of COVID-19 was positively associated with both preventive activity and likelihood of testing. Preventive activity was positively associated with likelihood of testing.

**Table 3:** Correlations between the main variables in Study 1

	1	2	3	4	5
1.Social support: access to help					
2.Self-efficacy	.43**				
3.Perceived risk of COVID-19	.08	−.13*			
4.COVID-19 preventive activity	.13	.07	22**		
5.Likelihood of testing for COVID-19	.17**	.10	.21**	.34**	

***p < .05 *p* < .01

#### The Impact of Socio-Economic Status Variables on Likelihood of COVID-19 Testing

First, a one-way ANOVA revealed that there was no significant impact of income group (<10,000, 10,000–19,999, 20,000–29,999, 30,000–39,999, 40,000–49,999, 50,000–59,999, >60,000) on the likelihood of COVID-19 testing (*p* > 0.05). An independent samples *t*-test revealed no significant impact of employment status (employed [*N* = 134] vs. unemployed [*N* = 93]) on the likelihood of COVID-19 testing (*p* > 0.05). Consequently, these variables were not examined further.

#### Multiple Regression Predicting Likelihood of COVID-19 Testing

A multiple stepwise regression was conducted with a bootstrap at 1000 samples to examine which variables predicted the variance of likelihood of COVID-19 testing. The variables of perceived risk of COVID-19, COVID-19 preventive activity, self-efficacy and social support – access to help were inserted as predictors, and likelihood of COVID-19 testing was inserted as the dependent variable.

COVID-19 preventive activity was entered into Step 1 and explained 11% of the variance in likelihood of COVID-19 testing. At Step 2, COVID-19 preventive activity and perceived risk of COVID-19 explained 13% of the variance in likelihood of COVID-19 testing. R-square change was 0.02 and F-change was 4.71 (*p* = 0.031). At Step 3, COVID-19 preventive activity, perceived risk of COVID-19, and social support – access to help explained 14% of the variance in likelihood of COVID-19 testing. R-square change was 0.02 and F-change was 4.05 (*p* < 0.05).

The regression model was statistically significant [*F*(3, 227) = 15.23, *p* < .001; *R*^2^ = 0.14]. Of all predictors, COVID-19 preventive activity with a β *=* 0.294 S.E. = 0.01, (*t* = 2.07, *p* < .001) was the most powerful, followed by perceived risk of COVID-19 with a β = 0.13, S.E. = 0.02, (*t* = 2.07, *p* < .05), and social support – access to help with a β = 0.13, S.E. = 0.02, (*t* = 2.01, *p* < .05). These findings support hypothesis 1.

#### Path Analysis

**Fig. 1:**
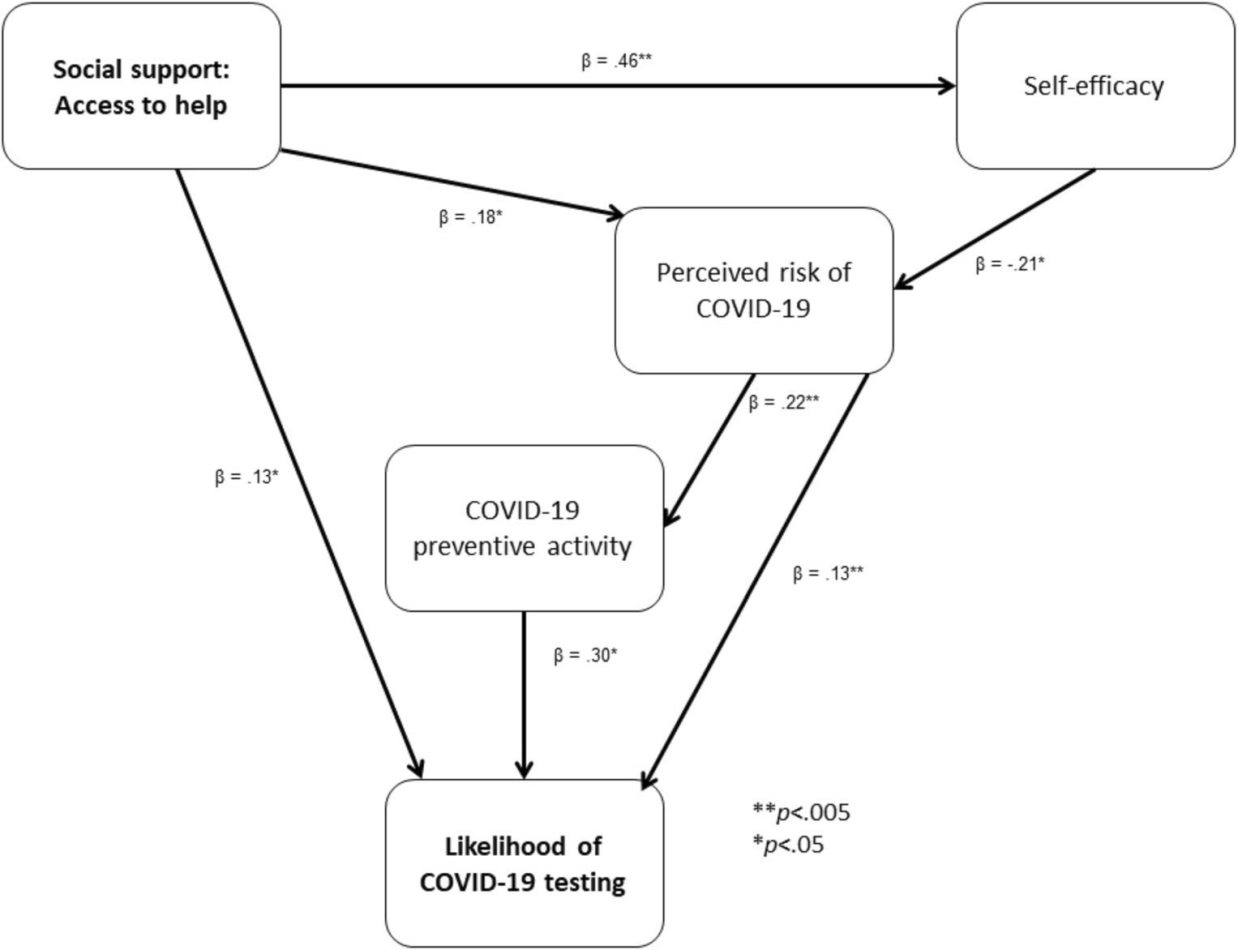
Path analysis of the relationship between social support: access to help and likelihood of COVID-19 testing through the mediators of self-efficacy, perceived risk of COVID-19, and COVID-19 preventive activity

Path analysis was performed using AMOS Version 20 with the main predictor of access to social support; and the mediators (self-efficacy; perceived risk; and preventive activity) to predict the dependent variable of likelihood of testing (see Fig. 1). A common method of testing the indirect effects is bootstrapping at 1000 samples (Shrout & Bolger, 2002). This is also recommended for correcting for relatively small sample sizes. The model fit was excellent with a Root Mean Square Error of Approximation (RMSEA) of .05; a Tucker-Lewis Index (TLI) of .95; and a Comparative Fit Index (CFI) of .99.

Social support – access to help had a main effect on the variance of likelihood of COVID-19 testing with a β = 0.13, S.E. = 0.02, *p* < 0.05. There were also mediation effects.

First, social support – access to help was positively associated with self-efficacy with a β = 0.46, S.E. = 0.13, *p* < 0.001, which in turn was negatively associated with perceived risk with a β = −0.21, S.E. = 0.06, *p* < 0.05. Perceived risk had a main effect on the dependent variable of likelihood of COVID-19 testing with a β = 0.13, S.E. = 0.16; *p* < 0.05. There were also mediation effects. Perceived risk was positively associated with preventive activity with a β = 0.22, S.E. = 0.08, *p* < 0.001, which in turn was positively associated with likelihood of testing [β = 0.30, S.E. = 0.01, *p* < 0.001].

Second, access to social support was positively associated with perceived risk [β = 0.18, S.E. = 0.05, *p* < 0.05], which, as described above, was related directly to likelihood of testing and indirectly through the mediator of preventive activity. These findings support hypotheses 2, 4 and 5, but do not support hypothesis 3.

## Study 2

The results of Study 1 indicated that access to social support (mediated by self-efficacy) was a significant predictor of likelihood of testing for COVID-19 after the effects of both perceived risk of COVID-19 and preventive activity were taken into account. Study 2 aimed to test the association between social support (this time, conceptualized in terms of neighborhood identification) and the corollaries of social support (strength of social network and decreased loneliness) on the likelihood of testing. Moreover, building on Study 1, the additional dependent variable of likelihood of vaccination against COVID-19 was introduced in Study 2 alongside that of likelihood of testing for the disease. The following specific hypotheses were tested:

1. Social support indexed in terms of neighborhood identification will be positively associated with strength of social network and negatively associated with loneliness.
2. Strength of social network will be negatively correlated with loneliness.
3. Social support via neighborhood identification will be positively associated with likelihood of COVID-19 testing and vaccination.
4. Loneliness will be positively associated with perceived risk of COVID-19.
5. Loneliness will be negatively associated with likelihood of vaccination against COVID-19.
6. Perceived risk of COVID-19 will be positively associated with preventive behavior and COVID-19 testing.
7. Preventive behavior will be positively associated with likelihood of both testing and vaccination.

### Method

#### Ethics

This project received ethics approval from Nottingham Trent University’s College of Business, Law and Social Sciences Ethics Committee. Participants provided electronic consent to participate, were debriefed and thanked for their time.

#### Participants

Two-hundred and fourteen residents in London completed an online survey on preventive activity during the COVID-19 pandemic. The survey was publicized by a South West London borough council on social media and by local charities and organizations that work in collaboration with the borough council. Participants were recruited in the first 3 weeks of September 2020. At that time, no vaccine was available and it was uncertain when one would become available. There were two sole eligibility criteria: (1) being aged 18 or over and (2) being a resident in the London borough. See Table 4 for a full summary of the socio-demographic characteristics of the participant sample in Study 2.

**Table 4:** Socio-demographic characteristics of the sample in Study 2

**Gender**	**Male**	**Female**						
	51 (23.8%)	163 (76.2%)						
**Citizenship**	**UK** *N* = 196 (91.6%)	**EU** *N =* 18 (8.4%)						
**Ethnicity**	**White British**	**White Other**	**Pakistani**	**Indian**	**African**	**Caribbean**	**Mixed**	**Asian Other**
	*N* = 151 (70.6%)	*N* = 41 (19.2%)	*N* = 2 (0.9%)	*N* = 3 (1.4%)	*N* = 6 (2.8%)	*N* = 2 (0.9%)	*N* = 6 (2.6%)	*N* = 3 (1.4%)
**Income**	**<£10,000**	**£10,000-19,999**	**£20,000-29,999**	**£30,000-39,999**	**£40,000-49,999**	**£50,000-59,999**	**>£60,000**	
	*N* = 33 (15.4%)	*N* = 40 (18.7%)	*N* = 37 (17.3%)	*N* = 37 (17.3%)	*N* = 18 (8.4%)	*N* = 6 (2.8%)	*N* = 43 (20.1%)	
**Education**	**Undergraduate**	**A−/ AS-levels**	**GCSE/O level**	**Postgraduate**	**Apprenticeship**	**Other**	**None**	
	*N* = 61 (28.5%)	*N* = 30 (14%)	*N* = 26 (12.1%)	*N* = 79 (36.9%)	*N* = 1 (0.5%)	*N* = 1 (0.5%)	*N* = 16 (7.5%)	

#### Measures

Participants were asked to indicate their age, gender, ethnicity, level of education, employment status and income.

##### Social Support: Neighborhood Identification

An adapted version of the positive affect and enactment items relating to identity (Vignoles et al., 2006) was used to measure social support via neighborhood identification. The scale consists of 5 items focusing specifically upon identification with Wandsworth where the study was conducted (e.g., ‘How important is being a Wandsworth resident in defining who you are?). The items were measured on a 5-point scale (1 = not at all important to 5 = extremely important). The scale manifested good reliability (α = .79).

##### Strength of Social Network

The Lubben Social Network Scale (Lubben et al., 2006) was used to measure strength of social network. The scale consists of 6 items (e.g.,‘How many relatives do you see or hear from at least once a month?). Items were measured on a 7-point scale (0 = none to 6 = 9 or more). The scale manifested excellent reliability (α = .89).

##### Loneliness

The 6-Item (Short) De Jong Gierveld Loneliness Scale (De Jong Gierveld & Tilburg, 2006) was used to measure loneliness. The measure consists of 6 items (e.g., ‘I miss having people around’), which were measured on a 5-point scale (1 = none of the time to 5 = all of the time). The scale manifested excellent reliability (α = .88).

##### Perceived Risk of COVID-19

The CORAS was used as in Study 1. The scale manifested excellent reliability (α = .84).

##### COVID-19 Preventive Activity

The CPBI was used as in Study 1. The scale manifested acceptable reliability (α = .68).

##### Likelihood of Testing for COVID-19 and Vaccination against COVID-19

The items ‘How likely is that, during the COVID-19 outbreak, that you will seek to get tested for the virus?’ and ‘…once it is available, get vaccinated against the virus?’ were measured on a 5-point scale (1 = extremely unlikely, 5 = extremely likely).

### Results

#### Descriptive Statistics

Table 5 provides an overview of the descriptive statistics for Study 2.

**Table 5:** Descriptive statistics for the key variables in Study 2

Continuous variables	Mean	SD	Minimum	Maximum
Age	58.20	13.85	29	89
Social support: neighborhood identification	14.14	3.74	5	24
Loneliness	16.83	5.16	6	29
Strength of social network	15.82	8.31	0	35
Perceived risk of COVID-19	19.57	4.34	7	30
COVID-19 preventive activity	40.52	6.04	15	50
Likelihood of testing for COVID-19	3.65	1.29	1	5
Likelihood of vaccination against COVID-19	4.18	1.23	1	5

#### Correlations

Table 6 provides an overview of the correlations between the main variables in Study 2. It is noteworthy that social support via neighborhood identification was positively associated with strength of social network and negatively correlated with loneliness. Strength of social network was negatively correlated with loneliness. These findings support hypotheses 1 and 2. Social support via neighborhood identification was positively correlated with likelihood of testing but not with likelihood of vaccination and thus hypothesis 3 was only partially supported.

**Table 6:** Correlations between the main variables in Study 2

	1	2	3	4	5	6	7
1.Social support: neighborhood identification							
2.Loneliness	−.19**						
3.Strength of social network	.22**	−.65**					
4.Perceived risk of COVID-19	.04	.19**	−.08				
5.COVID-19 preventive activity	.07	−.08	.05	.26**			
6.Likelihood of testing for COVID-19	.24**	.04	−.06	.38**	.32**		
7.Likelihood of vaccination against COVID-19	.04	−.18**	.21**	.14*	.32**	.24**	

***p < .05 *p* < .01

#### The Impact of Socio-Economic Status Variables on Likelihood of COVID-19 Testing

One-way ANOVAs revealed that there was no significant impact of income group (<10,000, 10,000–19,999, 20,000–29,999, 30,000–39,999, 40,000–49,999, 50,000–59,999, >60,000) on the likelihood of COVID-19 testing (*p* > 0.05) or vaccination (*p* > 0.05). Independent samples *t*-tests also revealed no significant effect of being in receipt of state benefits (yes [*N* = 42] vs no [*N* = 172]), as an additional indicator of socio-economic status, on likelihood of COVID-19 testing (*p* > 0.05) or vaccination (*p* > 0.05). Consequently, these variables were not examined further.

#### Multiple Regression Models

Multiple stepwise regressions were conducted with a bootstrap at 1000 samples to examine which variables predicted the variance of likelihood of testing and vaccination, respectively.

##### Predicting Likelihood of COVID-19 Testing

The variables of perceived risk of COVID-19, preventive activity and social support via neighborhood identification were inserted as predictors, and likelihood of testing was inserted as the dependent variable.

Perceived risk of COVID-19 was entered into Step 1 and explained 14% of the variance in likelihood of testing. At Step 2, perceived risk of COVID-19 and preventive activity explained 19% of the variance in likelihood of testing. R-square change was 0.05 and F-change was 14.03 (*p* < 0.001). At Step 3, perceived risk of COVID-19, preventive activity and social support via neighborhood identification explained 25% of the variance in likelihood of testing. R-square change was 0.05 and F-change was 12.87 (*p* < 0.001).

The regression model was statistically significant [*F*(3, 213) = 22.73, *p* < .001; *R*^2^ = 0.25]. Of all predictors, perceived risk of COVID-19 with a β *=* 0.32, S.E. = 0.02, (*t* = 5.068, *p* < .001) was the most powerful, followed by preventive activity with a β = 0.23, S.E. = 0.01, (*t* = 3.63, *p* = .001), and social support via neighborhood identification with a β = 0.22, S.E. = .02, (*t* = 3.59, *p* < .001).

##### Predicting Likelihood of COVID-19 Vaccination

The variables of perceived risk of COVID-19, preventive activity, strength of social network and loneliness were inserted as predictors, and likelihood of vaccination was inserted as the dependent variable.

Preventive activity was entered into Step 1 and explained 10% of the variance in likelihood of getting vaccinated. At Step 2, preventive activity and strength of social network explained 13% of the variance in likelihood of vaccination. R-square change was 0.04 and F-change was 9.25 (*p* = 0.03).

The regression model was statistically significant [*F*(2, 213) = 17.13, *p* < .001; *R*^2^ = 0.13]. Of all predictors, preventive activity with a β *=* 0.31, S.E. = .01, (*t* = 4.85, *p* < .001) was the most powerful, followed by strength of social network with a β = .19, S.E. = .01, (*t* = 3.04, *p* = .003).

#### Path Analysis

**Fig. 2:**
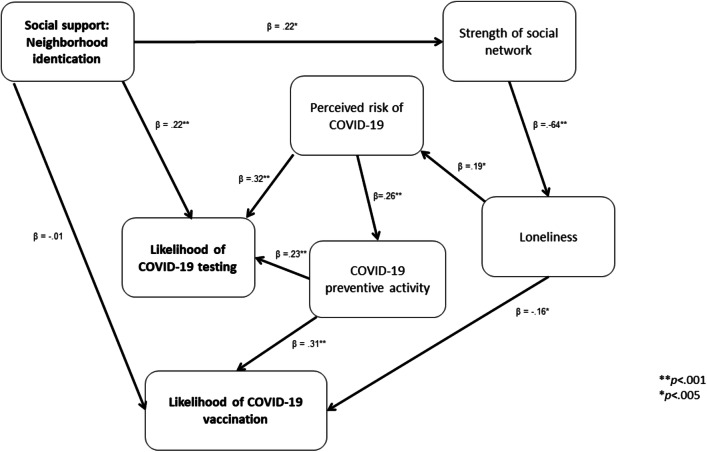
Path analysis of the relationship between social support: neighborhood identification and likelihood of COVID-19 testing and vaccination through the mediators of strength of social network, loneliness, perceived risk of COVID-19 and COVID-19 preventive activity

Path analysis was performed using AMOS Version 20 with the main predictor of social support - neighborhood identification; the mediators (strength of social network, loneliness, perceived risk of COVID-19 and COVID-19 preventive activity) to predict the dependent variables of likelihood of COVID-19 testing and COVID-19 vaccination, respectively (see Fig. 2). A bootstrap at 1000 samples was used. The model fit was excellent with a Root Mean Square Error of Approximation (RMSEA) of 0.07; a Tucker-Lewis Index (TLI) of 0.90; and a Comparative Fit Index (CFI) of 0.95.

Social support – neighborhood identification was not significantly associated with the dependent variable of likelihood of COVID-19 vaccination [*p* > 0.05]. It did have a main effect on the dependent variable of likelihood of testing with a β = 0.22, S.E. = 0.20; *p* < 0.001. There were also mediation effects.

First, social support - neighborhood identification was positively associated with strength of social network with a β = 0.22, S.E. = 0.15, *p* = 0.001, which in turn was negatively associated with loneliness [β = −0.64, S.E. = 0.03, *p* < 0.001]. Loneliness was positively associated with perceived risk of COVID-19 [β = 0.19, S.E. = 0.06, *p* = 0.004] which in turn was positively associated with likelihood of testing with a β = 0.32, S.E. = 0.02, *p* < 0.001. There was also a mediation effect. Perceived risk of COVID-19 was associated with increased COVID-19 preventive activity [β = 0.28, S.E. = 0.09, *p* < 0.001] which in turn was positively associated with likelihood of COVID-19 testing [β = 0.23, S.E. = 0.01, *p* < 0.001].

Second, as described above, social support - neighborhood identification was associated with strength of social network which in turn was related to loneliness. Loneliness was negatively associated with likelihood of COVID-19 vaccination with a β = −0.16, S.E. = 0.02, *p* = 0.014. There were also mediation effects. As described above, loneliness was associated with perceived risk of COVID-19 which in turn was related to preventive activity. COVID-19 preventive activity was positively associated with likelihood of vaccination [β = 0.31, S.E. = 0.01, *p* < 0.001]. These results provide partial support for hypotheses 1 and 3 and full support for hypotheses 2, 4, 5, 6 and 7.

## Discussion

In contrast to prior research findings (e.g., Neumann-Böhme et al., 2020), we found no significant effects of socio-economic variables (i.e., income, employment status, or receipt of state benefits) on the likelihood of COVID-19 testing or vaccination. This indicated that social psychological factors might provide a more important explanation of the variance in these variables. The present research builds on existing work (e.g., Yıldırım et al., 2020) by showing that perceived risk functions within a broader system of social psychological factors to determine one’s likelihood of testing and vaccination. In both studies, perceived risk of COVID-19 was directly associated with increased likelihood of testing and, in Study 2, indirectly with likelihood of vaccination. However, the social support variables (namely access to social support and neighborhood identification) and other aspects of social support (strength of social network and loneliness) constitute key components of this system of explanatory factors.

In Study 1, it was shown that social support was positively associated with perceived risk of COVID-19, possibly because within a supportive context it may be easier to acknowledge one’s risk, which has been shown to precipitate negative affect during the pandemic (Breakwell & Jaspal, 2020). Furthermore, people with higher social support (and thus higher levels of interaction with others) may be exposed to social representations of COVID-19 that enable them to appraise their risk more effectively. However, in Study 2, social support via neighborhood identification was not associated with perceived own risk of COVID-19 – only loneliness (as an aspect of decreased social support) was positively associated with perceived own risk. It is likely that the risk representations that are pervasive in the media and other channels of societal information are also accessible to people with higher levels of loneliness, promoting a high-risk perception in this group as well. However, the relationship between loneliness and perceived risk will need to be studied further.

In Study 1, the acquisition of social support (defined in terms of access to help in times of need) was in turn associated with greater likelihood of testing for COVID-19, which is understandable in view of the support that one would thereby have access to in the event of a positive test result. Indeed, it has been shown in previous research in other disease contexts, such as that of HIV, that, in the absence of social support, perceived risk of a disease may not result in self-protective behavior, including testing (Jones et al., 2019). Interestingly, Study 1 showed that self-efficacy mediated the relationship between social support and perceived own risk of infection, suggesting that increased self-efficacy makes it less likely that one will perceive oneself to be at risk of COVID-19. This was inconsistent with the hypothesis that self-efficacy and perceived risk would be positively correlated. Moreover, in the present data there was no significant relationship between self-efficacy and likelihood of testing, challenging existent work on the association between personal/collective resilience and self-protection (cf. Herrick et al., 2011; Zimmerman et al., 2013).

While in Study 1 social support was conceptualized as the availability of help from significant others in times of difficulty, in Study 2, it was defined in terms of neighborhood identification. Both facets of social support were associated with likelihood of testing – having help during times of difficulty and neighborhood identification were positively associated with likelihood of testing. However, in Study 2, social support was correlated only with likelihood of testing but not directly with likelihood of vaccination, which was not consistent with the hypotheses. However, neighborhood identification was indirectly associated with likelihood of vaccination through the other aspects of social support (i.e., greater social network and decreased loneliness). This may indicate that social support via neighborhood identification and strength of social network per se are not important determinants of likelihood of vaccination but rather that loneliness, in particular, is. This would appear to be consistent with research into likelihood of flu vaccination where it was found that those scoring high on loneliness were less likely to be vaccinated (Hajek & König, 2019). Those who are lonely may be less exposed to any positive social norms concerning vaccination. More generally, people with higher levels of loneliness have been found be less receptive to other healthcare services, such as cancer screening (Hajek et al., 2018). The important relation between loneliness and likelihood of COVID-19 vaccination will need to be studied further.

In short, in both studies, the pattern is the same – social support appears to constitute a direct or indirect determinant of likelihood of both self-protective behaviors. It is interesting that the failure/ absence of social support is strongly associated with subjective feelings of loneliness which, though negatively associated with perceived own risk of COVID-19, do appear to reduce the likelihood of vaccination. Loneliness was related to perceived risk of COVID-19 but not to preventive activity. These findings suggest that, though a corollary of social support, loneliness is a particular psychological state which should be considered in policymaking in relation to testing and vaccination as the pandemic progresses.

The results indicated that engaging in preventive activity was associated with increased likelihood of testing and vaccination. This suggests that, when individuals are already engaging in preventive activity, they may be primed to undertake additional self-protective activity, namely testing, which enables them to know their health status in relation to COVID-19, and vaccination, which, if available, would reduce their risk of infection with the virus. Those who perceive individual preventive behaviors, such as wearing a face mask and maintaining a physical distance from people outside of their household, to be socially normative in the fight against COVID-19 may also be more likely to endorse testing and vaccination. While Study 1 indicates that those who are less adherent to preventive activity are also less likely to test for COVID-19, Study 2 provides additional evidence that they are also less likely to seek vaccination. This would appear to be consistent with the concept of the ‘prevention norm’ which is shared and reinforced by members of a community (see also Jaspal et al., 2019) and with recent research showing the negative correlation between loneliness and vaccination likelihood due to decreased exposure to the social norm of vaccination (Hajek & König, 2019).

### Limitations

First, the relationship between loneliness and self-efficacy was not measured and how this may in turn shape likelihood of testing and vaccination was not examined. Second, the relationship between loneliness and perceived risk will need be studied further. Third, it would be advantageous to examine the impact of social contacts upon perceptions of testing and vaccination directly and how this in turn might shape likelihood of testing and vaccination. Fourth, vaccine hesitancy was not explicitly measured in these studies, which should be incorporated in future studies given the proliferation of misinformation and conspiracy theories in relation to the vaccine. Fifth, these cross-sectional studies do not allow us to ascertain causality and, thus, it is not possible to state unequivocally that decreased social support and increased loneliness make people less likely to test and to seek vaccination. These cross-sectional data should be triangulated using data from experimental and longitudinal studies in this area. Finally, it will be necessary to extend the present studies to non-Western countries, especially those with a collectivist cultural orientation (e.g., China and Vietnam) to examine the significance of social support to testing and vaccination likelihood in cultural contexts in which the social group is central to identity functioning (Wang et al., 2021). Furthermore, given the importance of family in collectivist societies, it would be beneficial to examine the impact of social support derived from within the household/ family context versus that from outside the family (Wang et al., 2021).

## Conclusions and Implications

Different measures of social support (availability of help in times of need and neighborhood identification) were administered to two different participant samples but the findings follow a similar pattern – social support appears to be associated with increased likelihood of testing (in both studies) and with vaccination (in Study 2). Extending the social cure perspective, which highlights the importance of social support from meaningful group memberships for coping behavior in response to psychological adversity, it is shown that distinct forms of social support may also prompt self-protective behaviors in response to the pandemic. The present studies show that the two measures of social support reveal different relationships with the important construct of perceived own risk of COVID-19, with only social support defined as availability in times of need, but not neighborhood identification, being positively associated with perceived own risk. Social support appears to facilitate preventive activity but the content of that support is important.

This research shows the nuances of social support that may be related to specific behavioral patterns associated with COVID-19 and, thus, the importance of considering distinct facets of this broad construct in research and policy in relation to disease prevention. Decreased perceived own risk may impede both preventive activity and likelihood of testing for the disease. While loneliness (as a psychological component of absence of social support) was positively associated with perceived own risk of infection, it does also appear to decrease the likelihood of vaccination.

The findings indicate that it will be important to demonstrate the value of testing and vaccination to individuals who are isolated, lonely and have decreased access to social support. Moreover, some tentative recommendations can be made for practitioners working with people at risk of decreased social support and loneliness. First, the results of these studies demonstrate additional negative sequelae of decreased social support, loneliness and isolation in the context of COVID-19 prevention – these factors are all associated, directly or indirectly, with decreased likelihood of COVID-19 testing and vaccination. Therefore, it is important to continue to invest in strategies and interventions that promote greater social support and connectedness in communities. Second, it has been shown that some groups (e.g., those facing health and socio-economic inequalities, older people) are at disproportionately high risk of social isolation and loneliness and thus these at-risk groups in particular should be given information, guidance and sign-posting in relation to both testing and vaccination (Jaspal & Breakwell, 2020). Third, although social support is an important correlate of testing and vaccination, it will also be necessary to ensure that accurate information about COVID-19 is circulated within the social groups that provide social support. This will be especially vital amid the uncertainty, mistrust and conspiracy theories that increasingly surround the COVID-19 pandemic.
